# Prevalence, risk factors and health-seeking behavior of menstrual disorders among women in India: a review of two-decade evidence

**DOI:** 10.1080/16549716.2024.2433331

**Published:** 2025-01-24

**Authors:** Puja Das, Suresh Jungari

**Affiliations:** a Department of Public Health and Mortality Studies, International Institute for Population Sciences, Mumbai, India; b Department of Public Health and Mortality Studies, Centre of Demography of Gender, International Institute for Population Sciences, Mumbai, India

**Keywords:** Menstrual disorders, risk factors, health seeking behavior, India

## Abstract

**Background:**

Menstrual health is critical for women of reproductive age. It is also evident that menstrual disorders have contributed to the increasing burden of non-communicable diseases.

**Objective:**

To our knowledge, no literature review explicitly addresses the prevalence, risk factors, and health-seeking behaviour of menstrual disorders in India. Therefore, the current study aims to synthesize the available scientific evidence on the prevalence and risk factors of menstrual disorders in India over the last two decades.

**Methods:**

We followed PRISMA guidelines to conduct the review. We used Google Scholar, PubMed, JSTOR, Scopus, and Sci Direct search engines to find eligible research studies and extracted data from 2000 to 2022. We also conducted quality appraisals of included studies in the review.

**Results:**

Results show that the prevalence of any menstrual disorders ranges from 3% to 87%. Among all menstrual disorders, Dysmenorrhea was reported to be high (46% to 76%) among women, followed by premenstrual symptoms (PMS) (40% to 71%), while PCOS (3% to 14.14%) was less. The study further found that irregular lifestyle, obesity, inadequate diet, age at marriage, family history, smoking, and place of residence factors is associated with menstrual disorders in India. As far as health-seeking for menstrual disorders is concerned, one-third of women sought treatment for menstrual disorders.

**Conclusion:**

The present study has revealed that most women reported high rates of Dysmenorrhea, while Polycystic Ovary Syndrome (PCOS) is less prevalent. The study findings suggest that health-seeking behaviour is the most important factor in reducing menstrual disorders, which has long-term effects of increasing other comorbidities.

## Paper Context

- **Main findings**: This literature review findings indicate that studies included in the review reported that more than half of the women have experienced one or more of the menstrual disorders. There is a great need to understand the menstrual disorders burden.
- **Added knowledge**: This review adds to the existing literature on menstrual disorders and their risk factors, such as sedentary lifestyle, obesity, inadequate diet, age at marriage, family history, smoking, and place of residence.
- **Global health impact for policy and action**: This review highlights that increasing menstrual disorders should be addressed in order to reduce the disease burden and associated negative outcomes. Further, interventions to improve the health-seeking behavior of menstrual disorders are needed to mitigate menstrual disorder impact on overall health.

## Background

Menstruation is a periodic phenomenon with an average cycle length of 21–34 days intervals [1,2]. The onset of menarche marks this transitional period, an important milestone in the ages of 10–14 years [2]. Menarche is characterized by the development of reproductive function and the impending end of physical growth [3]. Menstruation is vital in reproductive health and endocrine function [4]. Age at menarche may differ in different age groups, across geographical regions, cultural practices, and mother’s menarche history.

Recent emerging evidence shows that about 90% of women experiencing menstrual disorders are associated with their reproductive behaviour, comprising infertility, pregnancy and childbirth of women [5]. Menstrual disorders have become a widespread issue among women in their reproductive age. Worldwide, women’s increased gynaecological visits have been observed due to the culmination of menstrual disorders [5]. The menstrual disorder can be defined as an interruption in monthly cyclic order and the occurrence of some physical dysfunction related to menstruation [6]. Menstrual disorders include abnormal uterine bleeding (AUB), Dysmenorrhea, and premenstrual symptoms [7]. Previous studies in developing countries reported that the prevalence of abnormal uterine bleeding is 5% to 15% among young women in their reproductive age. This disorder is more prevalent among women aged 35 to 40 [8]. The frequency of amenorrhea ranges from about 5% to 9%, whereas irregular cycle was by 5% to 17%. Around 16% to 78% of women experience Dysmenorrhea, which is the most prevalent menstrual disorder [6].

Existing literature investigated that the aetiology of menstrual disorders was related to pathological factors, socioeconomic factors, urbanization status, nutritional status, lifestyle change, smoking behaviour, alcohol consumption, eating disorders, and menarche age [5,7,9]. Previous studies have found a range of risk factors for menstrual disorders, such as body mass index, which is associated with oligomenorrhea or amenorrhea. Studies on Dysmenorrhea reported that ages below 20 years, low body mass index, smoking, alcohol consumption, early menarche, prolonged flow and pelvic infection factors were associated with Dysmenorrhea [10]. Moreover, recent literature has emphasized that young adolescents and adult women in semi-urban and urban areas lead irregular lifestyles characterized by consuming processed foods and spending extended periods on digital screens. These habits play a critical role as risk factors in the occurrence of menstrual disorders [11–14].

Addressing the problems of menstrual disorders should be considered imperative in the larger domain of public health because it has severe medical consequences for women. The effect of menstrual disorders is not only limited to women’s reproductive age but also has health consequences beyond their reproductive age [8]. Past literature has reported that abnormal uterine bleeding can occur due to infection, fibroids, or cancer in the reproductive organs [15]. It has been found that irregular menstrual cycles could lead to polycystic ovary syndrome (PCOS). Long duration of experience of PCOS can result in infertility and has been a significant concern for many women in their reproductive age [16,17]. Furthermore, PCOS contributes to an increased risk of suffering from multi-morbidities in older ages [18]. Besides the medical consequences of menstrual disorders, PCOS often affects women’s lives and productivity. Dysmenorrhea can disrupt women’s daily activities and work schedules due to severe pain and uncontrollable relief management [19,20]. Moreover, disorders of the menstruation cycle can affect physical and psychological health among women in their reproductive lives, leading to low quality of life for women [21,22].

Among Low- and Middle-income countries (LMICs), healthcare seeking for reproductive health problems, particularly menstrual health-related problems, is low as it is perceived as a personal matter of women [23]. A study by Harlow & Campbell, 2004, found that only two-fifths of women sought to seek for menstrual complaints, and the rest, 60% of women, did not consult with doctors because they thought menstruation problems to be normal [6]. Recent literature on menstrual health was more focused on managing menstrual hygiene among adolescent school-going girls, as schools in developing countries lack proper washing facilities [24]. Menstrual unhygienic practices can lead to increased pelvic infection among adolescent girls [25]. This intense information regarding the consequences of menstrual disorders urges for adequate monitoring of menstruation as primary health care to reduce pelvic infections and anaemia.

### Need for the critical review

A Plethora of scientific evidence has been generated on India’s maternal and reproductive health issues [26]. When maternal mortality was high, the research focus was examining the risk factors of maternal deaths and providing policy inputs. India experienced a high Maternal Mortality Ratio (MMR), which drove research towards maternal health issues in the last century. Health issues driven by menstruation have received little attention due to more focus on maternal health. Further, available studies merely focused on age at menarche and its predictors [27]. Recently, public health researchers have been at the forefront of exploring varied dimensions of menstrual hygiene management, menstrual disorders, menstruation and culture [28] and the cultural and traditional influence of menstrual health in India [24]. Recent literature has also demonstrated a range of menstrual disorders prevalent among young women and their association with varied health outcomes. However, no systematic review is available to synthesize menstrual disorders in India. Therefore, there is a greater need to synthesize the literature on menstrual disorders in India in the last two decades.

### The objective of the critical review

- To study the prevalence of the menstruation disorder among women in India.
- To determine factors affecting menstrual disorders in women in India.
- To examine the health-seeking behaviour of menstrual disorders in India.

## Methods

### Method of searching an electronic database

The study used various electronic-based sources, such as Google Scholar, PubMed, JSTOR, Scopus, and Science Direct, to search for relevant literature. Keywords such as *menstrual disorders, menstrual diseases, menstrual health problems, health seeking for menstrual disorders, reasons for menstrual disorders, and menstrual-related health problems in India* were used while searching.

#### Inclusion criteria

For the critical review, research papers were selected with the following inclusion criteria-

1. Only those studies published in peer-reviewed journals
2. Studies reported the prevalence of menstrual disorders and health-seeking by women.
3. Studies focusing on different health summary measurements.
4. Articles published in English language only.
5. Studies published during the year 2000–2022.

#### Exclusion criteria

The exclusion criteria are –

1. Studies have inadequate sample sizes of less than 100.
2. Studies that are related to menstrual disorders in the Gynecological domain.

### Data extraction procedure

**Figure 1.**
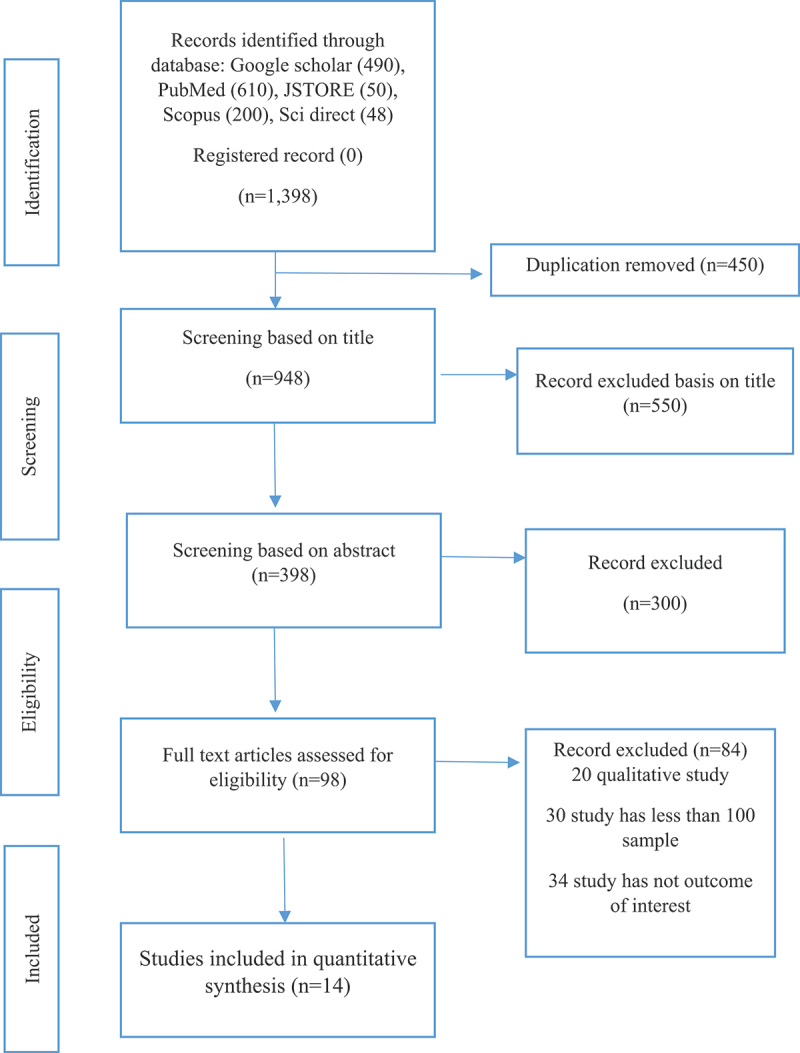
PRISHMA 2020 flow diagram of search and studies selected process.

Using the keywords mentioned above, the authors collected 1,398 papers from different databases, i.e. Google Scholar (*n* = 490), PubMed (*n* = 610), JSTOR (*n* = 50), Scopus (*n* = 200), Sci direct (*n* = 48). In the next stage, we used the end note software to identify duplicate studies, and after removing the duplicate, we had 948 studies. We have reviewed the titles of 948 studies and omitted 398 articles due to not meeting our study title. In the following stage, reading the abstract of all the selected articles, we found 98 articles for this systematic review. However, we have included 14 studies and removed the 84 articles based on <100 sample size (30), qualitative study (20) and 34 articles have not fulfilled the outcome interest in the review. The detailed flow chart (Figure 1) has been presented in the data extraction procedure.

**Table:** 

Menstrual disorders	Descriptions of symptoms
Dysmenorrhea	Continues pain during menstruation days
Oligomenorrhea	Infrequent occurrence of menstruation has more than 32 days
Polymenorrhea	Appearing of Menstruation in less than 21 days of the cycle
Pre Menstrual Syndrome (PMS)	A multi-symptom that occurs before the menstrual period, such as bloating or swelling of the abdomen, tender breasts, rapid mood change
Polycystic Ovarian Syndrome (PCOS)	When ovaries became immature or partially mature eggs in large numbers
Abnormal Uterine Bleeding (AUB)	Occurrence of Heavy bleeding during the periods or menopause phase
Uterine Cancer	Cancer forms in the uterus

### Quality appraisal of included studies

To include quality studies in the review, we have taken all possible steps to remove the sub-standard studies. It is crucial to have a singular quality checklist to maintain the high standards of the chosen research papers. Numerous checklists are available for assessing research study quality, and one of the most widely recognized tools in this regard is Boyle’s guidelines. Researchers commonly rely on Boyle’s guidelines to ensure the methodological rigour of included studies. These guidelines consist of eight criteria used to rate the methodological quality of the studies under consideration in this review. Each selected study is evaluated and assigned a score of 0 to 8 points. Any study scoring 2 points or less is categorized as failing to meet the required standard for methodological quality, resulting in its exclusion from the review [29]. For a comprehensive overview of the quality assessment of the included studies, go through Table 1.

**Table 1.** Quality appraisal of selected studies in the review.

Authors and year	Clearly defined population	Use of probability sampling	Response characteristics match the target population	Standardization of data collection method	Survey instruments reliable	Survey instruments valid	Features of sampling design accounted for in the analysis	Results included CI for statistical estimates	Score
Desai et al. [30]	Y	N	Y	Y	Y	Y	N	Y	6
Sarkar et al. [31]	Y	Y	Y	Y	Y	N	N	Y	6
Vidya et al. [32]	Y	N	Y	Y	Y	N	N	Y	5
Godbole et al. [21]	Y	N	Y	N	N	Y	N	N	3
Dhār et al. [33]	Y	N	Y	Y	Y	Y	N	Y	6
Sharma et al. [34]	Y	N	Y	Y	Y	Y	N	Y	6
Katole et al. [35]	Y	N	Y	N	Y	N	N	Y	4
Sharma et al. [36]	Y	N	Y	Y	Y	Y	N	Y	6
Negi et al. [37]	Y	N	Y	Y	N	N	N	Y	4
Kul Shrestha and Durrani. [38]	Y	N	Y	Y	Y	Y	N	Y	6
Singh et al. [39]	Y	N	Y	Y	N	N	N	Y	4
Lohani et al. [40]	Y	Y	Y	Y	Y	Y	N	Y	7
Sachan et al. [41]	Y	Y	Y	Y	Y	Y	N	Y	7
Ravi et al. [42]	Y	N	Y	Y	Y	N	N	Y	5

### Data extraction

From the selected studies in the review, the authors collected the following information-

- Author and year of publication
- Study design and sampling methods, study tools
- Prevalence rate of menstrual disorders
- Factors of menstrual disorders
- Health care seeking for menstrual disorders

### Data analysis

We have carefully selected papers for our systematic review, encompassing diverse research approaches aimed at investigating various aspects of menstrual disorders and risk factors of menstrual disorders. In this body of the literature, a significant knowledge gap is evident concerning the prevalence rates and causal factors behind menstrual disorders. Therefore, conducting a meta-analysis of the available data was found to be unfeasible, leading us to opt for a systematic review as our preferred research approach.

## Results

### Study characteristics

This review encompassed studies investigating the prevalence of menstrual disorders and the contributing factors. All the studies in this review adopted a cross-sectional study design, and their approach was exclusively quantitative. Within the reproductive age group of women, various menstrual disorders were examined. Across all the studies, a consistent finding emerged: menstrual disorders were more prevalent among younger and unmarried women, attributed mainly to their sedentary lifestyle. Study characteristics and summary are provided in Table 2.

**Table 2.** Summarized description of selected studies in the review.

Authors	Age Group	Sample size	State of India	Study type	Methods	Prevalence of Disorders	Co-factors	Health Seeking behaviours
Desai et al. [30]	13-18 years	881 school going girls	Gujarat	Cross-sectional study	Rotterdam criteria scale used. Questionnaire used for collecting the data.	This study found that 13.54% girls having PCOS.	Obesity, socioeconomic status, family history, insulin resistance are the risk factors of PCOS.	13.54% adolescent girls consulted with doctors
Sarkar et al. [31]	16-28 years	190 nursing students	West Bengal	Cross-sectional study	Semi-structured questionnaire	61.58% responds reported experiences of premenstrual syndrome.	Lack knowledge on reproductive health, low literacy rate, gender disparity, culture of silence factors affecting menstrual	31.05% of Nursing students visited to doctor’s chamber
Vidya et al. [32]	18-24 years	1068 young women	Tamil Nadu	Cross-sectional study	Rotterdam scale and questionnaire used for data collection.	The study population was 6% prevalence of PCOS in South India.	Leading factors of PCOS are place of residence and sedentary life style	20% young women have PCOS symptoms but never consulted with doctors in rural area.
Godbole et al. [21]	18-25 years	280 students	Maharashtra	Cross-sectional study	Pre-tested questionnaire	Prevalence of short bleeding periods, long bleeding periods were found 13.2% and 12.2% population. Dysmenorrhea was highly found about 68% girls. Only 3% girls documented PCOS.	Changing trends of life style, shifting dietary habit and tough competition factors are highly associated with menstrual problems.	———–
Dhar et al. [33]	10-30 years	420 urban adolescent	West Bengal	Cross-sectional study	Structured Questionnaire	Prevalence of PCOS, Dysmenorrhea, Menorrhagia, Polymenorrhea, Hypomenorrhea and the irregular menstrual cycle was found at 14.14%, 15.14%, 6.29%, 3.70%, 5.16%, and 44.83% respectively.	Risk factors of menstrual disorders were BMI, short sleep, unhealthy life style, sedentary and vigorous physical activities.	———–
Sharma et al. [34]	13-19 years	198 adolescent girls	Delhi	Cross-sectional study	Pre-tested questionnaire used.	About 67%, 63%, 31%, 14%, 7.1% of girls are having Dysmenorrhea, Premenstrual syndrome, irregular cycles, abnormal bleed, and missed cycle respectively.	—————	Only 4% adolescent girls have visited hospital regarding menstrual related problems.
Katole et al. [35]	15-49 years	570 women	Central India	Cross-sectional study and clinical based study	Pre-tested questionnaire was used.	This study found that about 8.9% is reported infertility in their reproductive age groups.	Socio-demographic factors such as risk factors associated with infertility age at marriage, nuclear family, higher education level, employed women, high socioeconomic status, obesity, irregular menstruation.	———-
Sharma et al. [36]	15-25 years	2673 women	Punjab	Cross-sectional study	Pre-designed questionnaire	Overall 60.61% women were suffering from menstrual problems, among of them Dysmenorrhea is most prominent about 50% where PCOS was 3%.	PCOS is related with unawareness from the irregular menstrual cycles.	Only 3.30% was reported to consult with doctors.
Negi et al. [37]	13-19 years	470 girls	Uttarakhand	Cross-sectional study	Questionnaire used	Dysmenorrhea (62.75%), premenstrual symptom (40%), irregular cycle (28.72%)	Irregular life style, food style change very much effect on menstrual disorders in young and adults females.	———–
Kulshrestha and Durrani. [38]	14-17 years	320 adolescent	Uttar Pradesh	Cross-sectional study	Pre-tested questionnaire	Overall menstrual disorders is recorded 76.9%. Highest is PMS of 71.3% followed by Dysmenorrhea 46.3%, amenorrhea 21.3 %, oligomenorrhea 12.8%, Polymenorrhea 22.2% respectively.	Irregular life style, and inadequate diet are risk factors of menstrual disorders. One solution is that physical exercise can reduce menstrual disorders.	———–
Singh et al. [39]	10-19 years	210 adolescence school going girls	Delhi	Cross-sectional study	Pre-tested and semi-structured questionnaire	Prevalence of Menorrhagia, irregular menses, Dysmenorrhea, Polymenorrhea are 6.6%, 5.7%, 76.1% and 5.2% respectively.	Age of menarche, family history factors associated with menstrual disorders	———–
Lohani et al. [40]	15-49 years	170632 women	India	Cross-sectional study	Pre-tested questionnaire	Dysmenorrhea-5.4%, irregular periods-4.2%	30-46 age group, illiterate women, young married women, lower socioeconomic background	———–
Sachan et al. [41]	10-19 years	847 adolescent girls	North Indian districts	Cross-sectional study	Structured interview schedule	Prevalence of Dysmenorrhea is highest among other menstrual problem about 73.7%. While 25% irregular menstrual cycle is reported.	Place of residence such as urban and rural area	———–
Ravi et al. [42]	10-19 years	350 school going girls	Tamil Nadu	Cross-sectional study	Interview scheduled of pre-tested questionnaire	In this they examined that 87.7% girls were suffered from menstrual problems. Highest prevalence of Dysmenorrhea is reported about 72% followed by menorrhagia and irregular menstrual cycles in 45.7% and 31.7% respectively.	Risk factors such as unawareness of menstrual problems, reluctance going hospital, lack of knowledge about menstrual absorbent in rural area.	———–

### Geographical distribution of included studies

Two studies were conducted in the western Indian states of Rajasthan, Gujarat, and Maharashtra. Six studies were carried out in northern India, in Punjab, Uttar Pradesh, Uttarakhand, and Delhi. Two studies were from the eastern part of the country, another one from the central region, and one study spanned multiple states across India. Two studies represented the Southern region of India. An analysis of the geographic distribution of these studies reveals that half of them were concentrated in the northern states of India. The highest menstrual disorders are found in the northern region of India.

### Prevalence of menstrual disorders

Our review mentioned a wide range of prevalence of menstrual disorders ranging from 60% to 80% [43,44]. Seven studies reported that Dysmenorrhea was a common problem among all menstrual problems and varied between 50% and 80% [19,37,38,42,44,45]. Four studies found that 40% to 71% of women have pre-menstrual symptoms. According to four studies, about 3% to 13% of the population have PCOS problems, and its prevalence rate is low compared to other menstrual problems [19,30,34,47]. One study found that about 13.2% and 12.2% of women suffered from short and long bleeding periods [19]. According to two studies, 21.3%, 12.8% and 3–22.2% of women complained about Amenorrhea, Oligomenorrhea and Polymenorrhea, respectively [42,32,46]. Two studies recorded that about 6–45.7% of girls have Menorrhagia [44,32]. All types of menstrual disorders are described in Table 3.

**Table 3.** Prevalence of different type of menstrual disorders (%).

	Menstrual disorders	Dysmenorrhea	Irregular cycles	PCOS	Amenorrhea	menorrhea	Polymenorrhea	PMS	Infertility
Sarkar et al. [31]	–	–	9.47%	–	–	–	–	61.58%	–
Sharma et al. [36]	60.61%	50%	–	3%	–	–	–	–	–
Katole et al. [35]	–	–	–	–	–	–	–	–	8.9%
Kulshrestha and Durrani et al. [38]	76.9%	46.3%	–	–	21.3%	–	22.2%	71.3%	–
Singh et al. [39]	–	76.1%	5.7%	–	–	6.6%	5.2%	–	–
Lohani et al. [40]	–	5.4 %	4.2%						
Desai et al. [30]	–	–	–	13.54%	–	–	–	–	–
Negi et al. [37]	–	62.75%	28.72%	–	–	–	–	40%	–
Vidya et al. [32]	–	–	–	6%	–	–	–	–	–
Dhar et al. [33]	–	15.14%	44.83%	14.14%		6.29%	3.70%	–	–
Ravi et al. [42]	87.7%	72%	31.7%	–	–	45.7%	–	–	–
Godbole et al. [21]	–	68%	–	3%	–	–	–	–	–
Sachan et al. [41]	–	73.7%	25%	–	–	–	–	–	–
Sharma et al. [34]	–	67%	31%	–	–	–	–	63%	–

### Factors associated with menstrual disorders

Factors associated with disorders of menstruation vary with region. Identifying risk factors behind menstrual disorders is one of the objectives of our systematic review study. Many studies have examined the socio-demographic characteristics associated with menstrual problems, such as age at marriage, nuclear family, higher education level, employed women, and high socioeconomic status [34,36]. Three studies explored that irregular lifestyle, sedentary lifestyle, inadequate diet, and unawareness of irregular menstruation are mentioned as risk factors associated with menstrual disorders in three studies [44,42,47]. Two studies identified that menarche age and family history often caused menstrual problems in the reproductive life span of women [22,48].

### Health seeking behaviors

Our research examined five of the fourteen studies that explored individuals’ tendencies to seek healthcare for menstrual issues. The prevalence of health-seeking behaviour exhibited regional disparities, with rates ranging from 4% to 31% [34,47,36]. These studies predominantly concentrated on PCOS, with one study revealing that 20% of young women exhibited PCOS symptoms but were hesitant to pursue treatment [36].

## Discussion

To this day, as far as we know, this is the first systematic review in India conducted to investigate the prevalence of menstrual disorders, associated factors, and behaviours related to seeking healthcare. Menstrual abnormalities entail a combination of one or more unfavourable symptoms linked to the menstrual cycle, and a similar occurrence rate of menstrual disorder was found in developed and developing countries [49]. We observed that the proportion of women suffering from at least one of the menstrual disorders ranges between 60% and 80%. Dysmenorrhea (50% to 80%) and Premenstrual Symptoms (40% to 71%) were the most widespread, while PCOS was less prevalent (3% to 13%). Hence, it is evident that menstrual disorders are widespread across India. A previous study indicated that more than 50% of women in the Middle East experienced menstrual disorders [50]. Among Italian adolescent girls, the occurrence rates of Dysmenorrhea, Polymenorrhea, Oligomenorrhea, Menorrhagia, and Irregular menstrual cycles were 6.2%, 3%, 3.4%, 19%, and 9%, respectively [8]. Among Iranian adult women, 41% of respondents reported Dysmenorrhea, while 22.1% dealt with irregular menstrual cycles [50]. A study in Australia focusing on teenagers revealed that 93% of adolescents endured severe pains and 96% experienced premenstrual symptoms [51]. Our findings align with research conducted in both developed and developing countries.

Our research also revealed that various factors are influencing menstrual disorders, with significant contributors being socio-demographic factors, age at menarche, family history, a sedentary lifestyle, dietary changes, obesity, excessive physical activity, limited understanding of reproductive health, and lack of awareness regarding menstrual issues [32,52]. Among the risk factors identified for menstrual disorders, lifestyle emerged as the most frequently cited factor in the studies analyzed for this review [44,42,47]. There is convincing evidence to support the idea that women who live erratic lives are more likely to suffer from menstruation illnesses.

Although previous studies have suggested that the occurrence of menstrual disorders and their influencing factors differs based on the place of residence, it has been observed that the prevalence of menstrual disorders is higher in urban areas compared to rural areas [41]. The prominence of PCOS and PMS cases is notably more significant in urban areas [53], likely due to the higher number of women engrossed in their professions with less time to prepare food and sustain their standard of living. It leads to subsequent alteration in dietary patterns as they often lean towards fast foods [18,36]. However, there has been a recent increase in the prevalence of PCOS disease in rural India, which can be attributed to the lack of awareness concerning reproductive health, traditional beliefs, and a tendency to avoid seeking medical check-ups [37]. On the other hand, Dysmenorrhea appears to be relatively similar in urban and rural areas [54].

Menstrual disorders exert an influence not solely on female reproduction or infertility but also on various other health aspects. Numerous morbidities have been linked to menstrual disorders, including conditions such as anaemia and thyroid dysfunctions [31]. Polycystic ovary syndrome (PCOS) represents a multifaceted endocrine and metabolic disorder characterized by insulin resistance, hyperandrogenism, and dyslipidemia, thus elevating the risk of associated comorbidities such as cardiovascular diseases, endometrial cancer, and type II diabetes [55]. This review further corroborates the noteworthy prevalence rate of PCOS within the spectrum of menstrual disorders.

In India, menstruation is associated with various rituals, practices, and cultural beliefs, and the sharing of menstrual information is primarily restricted among women and their mothers. Because of cultural barriers and a lack of openness about menstruation, many women hesitate to discuss their menstrual issues with healthcare professionals. Additionally, there is a significant knowledge gap among women regarding menstrual problems, primarily because women for generations perceive menstrual problems as normal [26]. Chouhan et al. conducted a study in Bihar and Uttar Pradesh. It revealed that about 33% of adolescent girls went to health professionals for menstrual disorders. Our study results corroborate previous studies, which found that health-seeking of menstrual disorders varies from 4% to 31%. However, knowledge about menstrual health is essential for reproductive well-being of women. Lack of knowledge on menstrual disorders may cause serious health complications in later life. In rural India, many girls may experience symptoms of conditions like PCOS but often do not seek treatment due to limited awareness about menstrual health issues.

In the specific context of India as a developing nation, government reproductive health policies tend to emphasize maternal health and pregnancy-related issues over broader gynaecological health concerns [54]. The National Rural Health Mission (NRHM) introduced a scheme in 2012 offering sanitary napkins at 6 rupees per packet in rural areas to encourage safer, more dignified menstrual practices [55]. However, most governmental efforts around menstruation focus on hygiene management rather than addressing menstrual disorders to promote comprehensive health and wellbeing. Implementing strategies like raising awareness through menstrual health education via social media and local and community-level camps should be contemplated to challenge prevailing stigmas associated with menstruation.

## Limitations of this study

Over one-third of the studies examined the prevalence of menstrual problems among adolescent and young adult females aged 15–25 years. All included studies used a cross-sectional study design, and most assessed the prevalence of menstrual problems and associated risk factors. The study results represent data from across India; however, there is a lack of studies from the northeast region of India, which is a limitation. This study did not include any qualitative studies, so some factors associated with menstrual disorders may not be reported in our review. Additionally, this study did not explore why health-seeking behaviour is low and did not examine the cultural factors associated with health-seeking behaviour. Furthermore, this research could not explain the consequences of menstrual disorders in adolescent girls and young adults, such as anaemia and infertility, nor whether they affected other comorbidities during menopause or late reproductive age.

## Conclusion

Our review examined the prevalence of menstrual disorders, risk factors associated with these disorders, and health-seeking behaviour in India. Socioeconomic factors, educational level of women, sedentary lifestyle, dietary pattern, obesity, stress, smoking, place of residence, and BMI are important factors associated with menstrual disorders. The review reported a higher prevalence of Dysmenorrhea; however, PCOS problems were less prevalent, which may be due to under-reporting [36,54]. Our review indicates that high levels of Dysmenorrhea are particularly prevalent among girls, affecting their daily activities and quality of life. Furthermore, health-seeking behavior is the most important factor in reducing menstrual disorders. Identifying and treating early can prevent the development of other comorbidities such as infertility, diabetes, and cardiovascular diseases. Our study suggests that promoting education and awareness campaigns regarding menstrual health, improving access to healthcare facilities, and encouraging healthy lifestyle choices can significantly reduce the prevalence of menstrual disorders.
